# Amyloid PET Disclosure in ADNI‐4: The Clinician Experience

**DOI:** 10.1002/alz70857_106634

**Published:** 2025-12-26

**Authors:** Kristin Harkins, Jason Karlawish, Kyra O’Brien, Claire M Erickson, Josh D Grill, Susan M. Landau, Monica G Rivera Mindt, Ozioma C. Okonkwo, Ronald Petersen, Paul S. Aisen, Michael S. W. Weiner, Emily A. Largent

**Affiliations:** ^1^ University of Pennsylvania Perelman School of Medicine, Philadelphia, PA, USA; ^2^ Banner Alzheimer's Institute, Phoenix, AZ, USA; ^3^ The UC Irvine Institute for Memory Impairments and Neurological Disorders, Irvine, CA, USA; ^4^ Neuroscience Department, University of California, Berkeley, Berkeley, CA, USA; ^5^ Fordham University, New York, NY, USA; ^6^ Wisconsin Alzheimer's Disease Research Center, School of Medicine and Public Health, University of Wisconsin‐Madison, Madison, WI, USA; ^7^ Department of Neurology, Mayo Clinic, Rochester, MN, USA; ^8^ Alzheimer's Therapeutic Research Institute, University of Southern California, San Diego, CA, USA; ^9^ University of California, San Francisco, San Francisco, CA, USA; ^10^ University of Pennsylvania, Philadelphia, PA, USA

## Abstract

**Background:**

Prior studies of Alzheimer's disease (AD) biomarker disclosure have answered important questions about individuals’ experiences learning amyloid results. In comparison, the experiences of individuals *performing* AD biomarker disclosure have been under characterized. The present study examines the clinician experience disclosing amyloid positron emission tomography (PET) results within the Alzheimer's Disease Neuroimaging Initiative's ADNI‐4 study.

**Methods:**

The ADNI‐4 longitudinal cohort study includes a pragmatic process for amyloid PET disclosure for participants across the cognitive spectrum. Disclosing clinicians complete a short questionnaire after each disclosure session, reporting on the visit length, the clinician's rating of participant and study partner understanding and emotional reactions, topics discussed, challenges, assessment of the visit overall, and any feedback about the process.

**Results:**

Across the first 100 disclosures (48 cognitively normal, 35 Mild Cognitive Impairment (MCI), 17 dementia) in ADNI‐4, visits lasted an average of 29.4 minutes (SD 13.9, range 10‐90). Most cognitively impaired participants had a loved one present at the disclosure visit (dementia 94%, MCI 80%) but the majority of cognitively normal participants (73%) did not.

Clinicians rated understanding of the result as adequate for all study partners and the vast majority (94%) of participants. Clinicians reported feeling “very confident” in their ability to assess readiness to receive result (70%), disclose (73%), and answer questions (77%). Clinicians rated all visits as “good” or better, with two‐thirds (65%) rated as “excellent.”

Frequently discussed topics included meaning of result (99%), other risk factors (72%), and emotional impact of result (49%). Stigma and discrimination were rarely discussed, coming up in 12% and 3% of disclosure sessions, respectively. Challenges were infrequent (7%) and primarily included desire for additional information and challenges related to the participant's impairment. Clinicians provided the study team feedback about the disclosure process for 25% of disclosure visits. Comments included the desire for additional information or resources, positive feedback, and the importance of the study partner.

**Conclusions:**

Clinicians disclosing amyloid PET results in ADNI‐4 report high confidence, adequate participant and study partner understanding, and few challenges. These data from clinicians, in combination with participant data, will inform future disclosure practices in both research and care.

